# The Characteristics of Training Injuries Among Chinese Competitive Rugby Sevens Players: A Retrospective Study

**DOI:** 10.3390/healthcare14111548

**Published:** 2026-06-02

**Authors:** Shuxuan Li, Ziwen Mu, Hanyan Yan, Qiran Li, Kazuhiro Imai, Shaoshuai Shen, Cheng Liu, Lin Zhang, Xiao Zhou

**Affiliations:** 1 School of Physical Education, Huazhong University of Science and Technology, Wuhan 430074, China; u202317088@hust.edu.cn (S.L.); m202475613@hust.edu.cn (Z.M.); m202475611@hust.edu.cn (H.Y.); u202317072@hust.edu.cn (Q.L.); fatimaliu@hust.edu.cn (C.L.); 2 Department of Life Sciences, Graduate School of Arts and Sciences, The University of Tokyo, Tokyo 153-8902, Japan; imai@idaten.c.u-tokyo.ac.jp; 3 School of Education and Welfare, Aichi Prefectural University, Nagakute 480-1198, Japan; shin-shosui@ews.aichi-pu.ac.jp

**Keywords:** competitive athletes, Rugby sevens, sports injuries, injury surveillance, age-specific

## Abstract

**Highlights:**

**What are the main findings?**

- Among 414 Chinese rugby sevens players aged 14–24 years, the overall training injury rate is 0.80 per 1000 training hours, with the ankle and knee being the most common injury sites.
- Players > 18 years have a significantly higher injury rate than those ≤18 years, and female players have a higher injury rate than male players, especially females > 18 years (1.41 per 1000 training hours).

**What are the implications of the main findings?**

- In the study, we clarify the age- and gender-specific distribution characteristics and high-risk injury sites of rugby sevens training injuries in young Chinese players, providing key epidemiological data.
- The findings lay a foundation for formulating targeted injury prevention and rehabilitation programs based on age and gender, thereby improving the safety of rugby sevens.

**Abstract:**

**Background**: Rugby sevens has developed rapidly in China, but research on its related injuries remains limited; thus, clarifying injury characteristics is crucial for targeted prevention. **Objective**: The aim of this study is to investigate the distribution and incidence of training-related rugby sevens injuries by age and gender. **Methods**: A cross-sectional retrospective study was conducted using a self-reported questionnaire, recruiting 414 competitive rugby sevens players (aged 14–24 years) who participated in competitions at the city level or higher. Injuries were defined as rugby-related physical discomfort with training time loss, or medical care regardless of time loss. Injury rates were calculated via the Poisson distribution as standardized incidence per 1000 training hours. **Results**: Among 414 participants, 141 reported 283 injuries, with an overall rate of 0.80/1000 h (95% CI: 0.71–0.90), and the ankle (20.1%) and knee (18.7%) were the most common sites. The participants > 18 years had a higher injury rate (1.21/1000 h, 95% CI: 1.03–1.38) than those ≤18 years (0.52/1000 h, 95% CI: 0.42–0.62). Females (1.00/1000 h, 95% CI: 0.82–1.19) had a significantly higher rate than males (0.70/1000 h, 95% CI: 0.60–0.81), with females > 18 years having the highest rate (1.41/1000 h, 95% CI: 1.12–1.71). **Conclusions**: Lower-limb injuries (ankle, knee) are most common in 14–24-year-old elite players. Participants > 18 years and females have higher injury risks, providing epidemiological data for age- and gender-specific prevention.

## 1. Introduction

Rugby is a rapidly growing, high-intensity collision sport played worldwide, with member unions in 132 countries and approximately 8.46 million participants [1]. However, its technical and physical demands including ball carrying, rapid acceleration and deceleration, and high-speed changes in direction, together with frequent collisions and tackles, place players at substantial risk of injury. Rugby-related injuries not only hinder athletic performance but also represent a major factor limiting career longevity and the sustainable development of athletes [2].

Previous epidemiological studies have consistently demonstrated the high injury burden associated with rugby during matches. Given that we focus solely on training-related injuries in this study, a detailed distinction between injury incidence rates in training and match environments was made. In elite rugby sevens training, female players sustained 1.0 to 2.2 injuries per 1000 training hours [3], while this value for male players was 0 to 1.2 [4]. By contrast, match play imposes substantially higher risks with the overall injury incidence ranging from 101.5 to 119.8 injuries per 1000 player-match hours. Even among amateur players, whose contact intensity is lower, the match injury incidence remains high at 74.7 injuries per 1000 player-match hours [5,6]. Sports injuries have become one of the main causes of athletes’ retirement, with 36% of rugby players ending their careers due to injury [7]. With respect to injury mechanisms, tackling (23.4–35.4%), ball carrying (16.1%), and contact-related falls (8.8–13.8%) are the most common injury-associated actions [3,8]. Among these, tackling is the most frequent and hazardous contact event in training, accounting for approximately two-thirds of all injuries [9]. Lower-limb injuries are particularly prominent in rugby training. The sport requires repeated cutting, turning, and twisting under physical contact during training, subjecting the lower extremities to sustained non-physiological mechanical loading and thereby markedly increasing training injury risk [10]. Although the incidence rate of training-related knee injuries is low, about 0.2 injuries per 1000 training hours, recent evidence indicates that this value ranges from 9.3 to 10.3 in men’s rugby union matches [11,12]. Moreover, that of ankle injuries in matches ranges from 6.4 to 9.7 [12,13]. The knee and ankle are therefore among the most commonly injured lower-limb joints in both male and female athletes during training. Notably, non-contact knee injuries in female players remain persistently prevalent during training, largely owing to repetitive joint loading and frequent directional changes during training sessions [13].

In addition to lower-extremity injuries, upper-extremity injuries, especially shoulder injuries, represent a major concern in rugby training. The most common mechanism of shoulder injury was tackling, which accounts for 67.6% of all shoulder injuries and was also the primary mechanism leading to shoulder dislocation [12]. As the shoulder undergoes direct impact and explosive loading during high-speed collisions, this region is highly vulnerable to rotator cuff tears and elevated injury risk. The incidence of shoulder injury also differs substantially by setting: during training, the rate is only 0.10 per 1000 player-hours, whereas this increases to 8.90 during competition [14]. Once injured during training, restricted shoulder mobility directly impairs basic upper-extremity functions such as arm elevation and weight bearing, thereby compromising subsequent training and competitive performance. Furthermore, inadequate scientific training regimens and limited medical support may further elevate injury risk and delay recovery. The high recurrence rate of sports injuries not only increases the acute health burden but may also exert long-term adverse effects on athletes’ health. This issue is particularly prominent in young rugby players, who face a higher risk of recurrent and secondary injuries due to limited access to specialized medical care [15].

Taken together, the high incidence and burden of injury in rugby have become major constraints on the healthy development of the sport, so it is necessary to clarify the injury characteristics of elite rugby players. Rugby sevens-related injuries caused by the characteristics (e.g., tackling, ball carrying) of Western populations are well understood; however, Chinese populations may present differentiated injury epidemiological characteristics due to unique domestic training modes. Nonetheless, current epidemiological evidence on Chinese competitive rugby sevens players remains limited. Therefore, we employed an epidemiological survey in this study to investigate the age- and gender-specific characteristics of training-related injuries in Chinese competitive rugby sevens players. The findings aimed to provide evidence-based data to help Chinese coaches and sports medicine specialists develop targeted injury prevention programs, to improve player safety levels, and promote the sustainable development of rugby in China.

## 2. Materials and Methods

During January–April 2025, a retrospective cross-sectional survey was conducted via self-reported questionnaires. Participants were recruited through convenience sampling from formal rugby teams affiliated with provincial and municipal sports authorities, including high-performance elite squads from Tianjin, Shandong, and Hunan that regularly participate in high-level national competitions. All rugby athletes completed anonymous questionnaires either online or offline during major official events, including the National Rugby Sevens Championship and the National Youth (U-Series) Rugby Sevens Championship. Offline data collection was performed by distributing and collecting printed paper questionnaires at competition venues, while online surveys were administered by sending standardized electronic questionnaire links to the official social media accounts of athletes. For adolescent participants under 18 years of age, questionnaire completion was completed under mandatory guardian supervision to ensure ethical compliance. Prior to formal enrollment, all participants were fully informed of the research objectives, survey content, and standardized completion guidelines. Written informed consent was obtained from every athlete, and parental informed consent was additionally acquired for minor participants.

Paper questionnaires were distributed to athletes during the interval periods before, during, or after matches, with a minimum 1-h rest interval to avoid interference with competitive status. To reduce reporting bias, the specific research hypotheses were not disclosed to participants; only the general purpose of investigating rugby-related sports injuries was explained. All questionnaire items were designed with neutral wording, and participants were required to fill in responses strictly based on their actual training and competition experiences. For online recruitment, formal written permission was granted by event organizing committees before distributing electronic survey links. Participants independently completed the online questionnaire via the shared link, and all response data were automatically captured, encrypted, and stored in the system upon submission. In total, 447 rugby athletes were initially recruited, and after strict inclusion and exclusion screening, 414 eligible participants were included in the final statistical analysis.

Predefined inclusion criteria were as follows: (1) a minimum of one consecutive year of formal rugby training experience; (2) regular participation in no fewer than two weekly training sessions; (3) absence of ongoing sports-related injuries and no history of orthopedic or sports surgery; and (4) valid questionnaire responses without logical errors or outlier data.

The questionnaire consisted of three standardized sections. The first section collected basic demographic and training-related information, including age, height, body weight, national sports grade certification, total rugby training years, weekly training frequency, single-session training duration, and monthly training weeks. The second section focused on injury surveillance, covering routine pre-training warm-up, the occurrence of rugby-related injuries, and specific injury scenarios (e.g., skill drills, resistance training, and daily auxiliary training). Participants retrospectively reported all injuries sustained over the preceding 12 months, with detailed information on injured body regions, onset time, recovery days, and post-injury intervention measures. Standardized anatomical diagrams were attached to the questionnaire with unified operational guidelines, assisting participants in accurately identifying injured or physically uncomfortable body sites that required medical intervention or restricted sports participation. A total of 25 anatomical regions were investigated: the face, chest, abdomen, shoulder, elbow, wrist, palm/finger, groin, quadriceps, knee, shin, ankle, toe, head, neck, scapula, back, lower back, buttocks, upper arm, forearm, hamstrings, calf, Achilles tendon, and plantar.

## 3. Injury Definition

The injury definition applied in this study was adapted from international consensus guidelines for injury surveillance in rugby sevens.

Injuries were categorized into two mutually exclusive types:

(1) Time-loss injury: Any physical complaint sustained during rugby training or competition that resulted in a player being unable to fully complete scheduled training or match activities within 24 h.
(2) Medical-attention injury: Any rugby-related physical discomfort requiring professional medical evaluation or treatment, regardless of subsequent time loss from participation. This dual-category definition simultaneously captures time-loss and medical-attention outcomes, consistent with standardized sports injury reporting frameworks [16].

**Figure 1:**
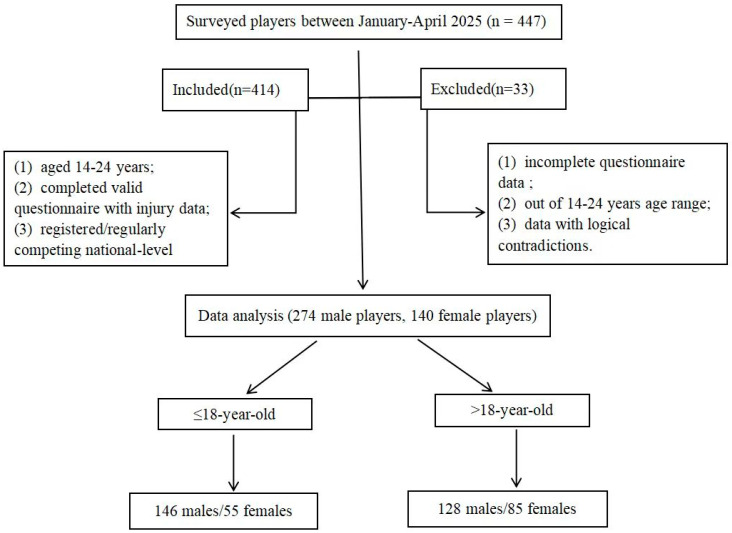
Screening flow.

The participant screening flow is displayed in Figure 1. Among the initial 447 surveyed participants, 33 (7.38%) were excluded from the final analysis, with reasons including incomplete questionnaire data (n = 17, 3.80% of the total sample), age outside the predefined 14–24-year range (n = 9, 2.01%), and logically contradictory data records (n = 7, 1.57%). A total of 414 participants met the criteria (140 females, 274 males). In addition, the number of participants aged >18 years (201 participants) was close to that of those aged ≤18 years (213 participants), so 18 years was set as the cutoff value. As shown in Figure 1, all participants were stratified into subgroups based on age and sex for subsequent data analysis.

Ethical approval for this study was obtained in line with the Declaration of Helsinki. Written informed consent was secured from each participant, and from the guardians of those who were minors.

## 4. Statistical Analysis

The normality of baseline variables was assessed using the Shapiro–Wilk test. Height was found to follow a normal distribution, whereas age, weight, BMI, years of training experience, daily training hours, weekly training days, weekly training hours, and annual training hours did not (all *p* < 0.05). Participants were divided into two age groups: ≤18 and >18 years. Between-group comparisons of baseline characteristics were performed using the independent-samples *t*-test for normally distributed data and the Mann–Whitney U test for non-normally distributed data.

Injury rates were calculated per 1000 training hours and per 1000 training sessions based on the Poisson distribution, with 95% confidence intervals (CIs). One training hour was defined as one hour of participation in training, and one training session was defined as a day on which an athlete engaged in training. The injury rate per 1000 training hours was calculated as:

Injury rate per 1000 h = {∑(total injuries)/∑[(daily training hours × weekly training days × annual training weeks per participant)]} × 1000

The injury rate per 1000 training sessions was calculated as:

Injury rate per 1000 sessions = {∑(total injuries)/∑[(weekly training days × annual training weeks per participant)]} × 1000

The denominator in both formulas represents the total training hours or sessions summed across all participants. To determine whether injury rates differed significantly between groups, non-overlapping 95% CIs were considered as indicating a statistically significant difference.

Logistic regression models were used to examine the association between rugby-related injury occurrence and potential risk factors. Three models were constructed with progressive adjustment: Model 1 adjusted for gender and age; Model 2 additionally adjusted for height and BMI; Model 3 further included years of training experience, daily training hours, and weekly training hours. All regression results were reported as odds ratios (ORs) with 95% CIs. A two-tailed *p*-value < 0.05 was considered statistically significant. The variance inflation factor (VIF) was calculated to diagnose collinearity. A VIF ≥ 10 indicated significant collinearity, in which case only one variable from a collinear pair was entered into a given model.

## 5. Results

A total of 414 participants met the inclusion criteria, including 274 males and 140 females (Table 1). Among male participants, 86 reported at least one injury, accounting for 165 injury cases, whereas among female participants, 55 reported at least one injury, accounting for 119 injury cases. The corresponding proportions of injured athletes were 31.4% in males and 39.3% in females.

Table 2 shows significant between-sex differences in age (*p* < 0.001), weight (*p* < 0.001), years of experience (*p* < 0.001), training times per week (*p* = 0.003), training hours per day (*p* = 0.014), training hours per week (*p* < 0.001), and training hours a year (*p* < 0.001).

**Figure 2:**
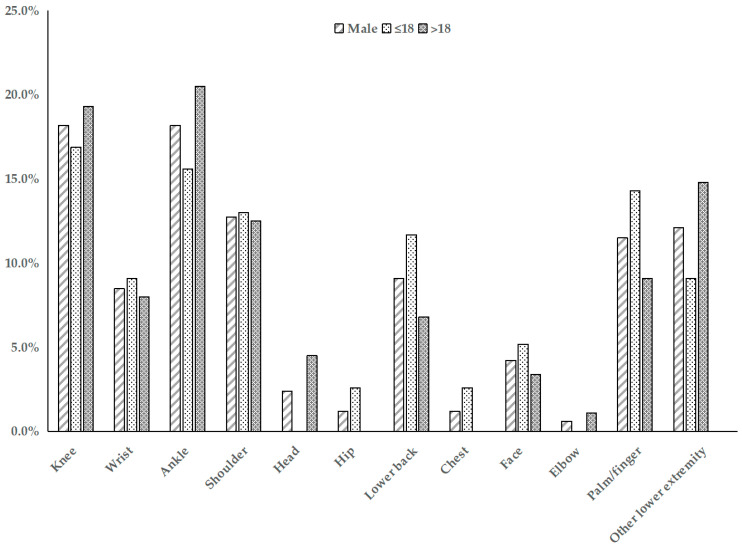
Occurrence of injuries in male rugby athletes.

**Figure 3:**
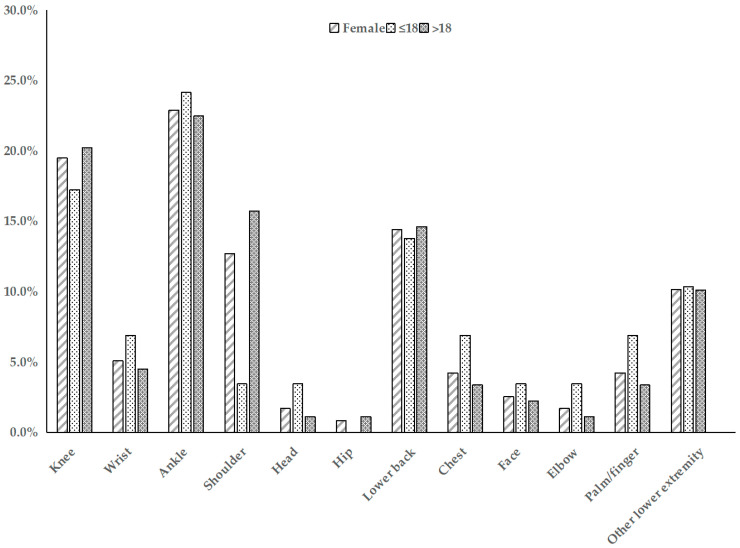
Occurrence of injuries in female rugby athletes.

Among all participants, the ankle was the most frequently injured site (20.1%), followed by the knee (18.7%), shoulder (12.7%), and lower back (11.3%) (Table 3). By sex, the knee (18.2%) and ankle (18.2%) were the most common injury sites, followed by the lower back (10.3%) and wrist (8.5%) in male athletes (Figure 2). In female athletes, the ankle (22.9%) was the most common injury site, followed by the knee (19.5%), shoulder (15.3%), and lower back (12.7%) (Figure 3). In both sexes, athletes > 18 years showed a higher proportion of injuries than those ≤18 years.

The injury rates per 1000 training hours by age, gender, and anatomical site are shown in Figure 2 and Figure 3. Among male athletes, the proportion of ankle injuries was higher for those >18 years (20.5%) than ≤18 years (15.6%), while palm/finger injuries were more frequent for ≤18 years (14.3% vs. 9.1%). Among female athletes, the proportion of shoulder injuries was higher for >18 years than ≤18 years (15.7% vs. 3.5%), while ankle and knee injuries remained at relatively high proportions for >18 years (22.5% and 20.2%, respectively). Notably, lower-back injuries in female athletes were slightly higher for >18 years (14.6%) than ≤18 years (13.8%), whereas male athletes showed an opposite trend (11.7% in ≤18 years vs. 6.8% in >18 years).

Table 4 shows injury rates per 1000 training hours by anatomical site for all participants by age. Overall, the total injury rate was 0.80 injuries per 1000 training hours (95% CI: 0.71–0.90). Female athletes exhibited an overall injury rate of 1.00 injuries per 1000 training hours, which was significantly higher than that of male athletes (1.00, 95% CI: 0.82–1.19 vs. 0.70, 95% CI: 0.60–0.81). Athletes aged >18 years had a significantly higher injury rate than those aged ≤18 years (1.13, 95% CI: 0.95–1.30 vs. 0.45, 95% CI: 0.35–0.55).

As shown in Table 5, male participants aged >18 years sustained 1.05 injuries (95%: 0.83–1.27) per 1000 training hours, which was significantly greater than the rate in male participants aged ≤18 years (0.39, 95% CI: 0.27–0.50). Similarly, female participants aged >18 years sustained 1.41 injuries (95% CI: 1.12–1.71) per 1000 training hours, which was significantly higher than that in female participants aged ≤18 years (0.41, 95% CI: 0.22–0.60).

Using collinearity diagnostics, the VIF value between height and BMI was 1.000, and that between training years of experience and BMI was 1.000; hence, BMI, height, and training years of experience were all used for analysis. The logistic regression results show that rugby-related injury incidence was significantly associated with gender (OR: 2.03, 95% CI: 1.00–4.14, *p* = 0.050) and age (OR: 1.14, 95% CI: 1.03–1.27, *p* = 0.013) (Table 6).

## 6. Discussion

In this study, we conducted an epidemiological survey of injuries among rugby sevens union players aged 14–24 years in China. More than one-third of the athletes had experienced at least one rugby-related injury. The injuries frequently occurred in the lower extremities, especially the ankle, followed by the knee and shoulder. The injury rate per 1000 training hours was significantly higher in athletes aged >18 years than in those aged ≤18 years. Female athletes aged >18 years also had a significantly higher injury rate than males in the same age group. This indicates that sex differences in injury risk persist regardless of age. Therefore, injury prevention programs for rugby sevens should be designed separately for training and match environments. These two settings differ in physical demands, exposure patterns, and injury mechanisms. In training settings, preventive strategies should focus on structured load management, progressive adaptation, and targeted neuromuscular conditioning. These approaches aim to improve musculoskeletal resilience, standardize movement patterns, and reduce overuse injuries.

By contrast, match prevention protocols must address acute high-intensity loading, fatigue accumulation during competition, and collision-related trauma. Emphasis should be placed on real-time risk control and proper tackling technique. Preparedness for acute injuries under unpredictable, high-stakes conditions is also essential [17,18]. The overall injury rate in our study was 0.8 per 1000 training hours. This is substantially lower than the 43.2 reported for international elite rugby sevens players, and also lower than average rates from related studies (108.5 for males, 76.1 for females) [5]. This discrepancy is likely due to differences between training and match environments. Training sessions are typically controlled and structured for skill or conditioning development. In contrast, competitive matches involve much higher physical intensity, aggressive maneuvers, and unpredictable movements. Previous two-year follow-up studies on match injuries have shown that players are pushed to their physiological limits. This leads to a higher risk of acute musculoskeletal trauma compared with the safer, more predictable nature of supervised training. We confirmed in this study that the knee and ankle were the most common injury sites among rugby union players, which agrees with previous international studies [6,19,20]. An analysis of rugby events at the 2016 Rio Olympic Games showed that ligament injuries involving the ankle and knee accounted for 35.2% of all injuries in female athletes, significantly higher than the 28.7% observed in male athletes [4]. Another study reported that lower-extremity injuries accounted for 63.2% of all injuries in female rugby players, with the knee (13.2%), ankle (12.5%), and thigh (11.2%) being the common sites [21]. Consistent with these reports, we found that injuries in female athletes were concentrated mainly in the knee, ankle, and shoulder in this study, with lower-extremity injuries accounting for the largest proportion [6]. Furthermore, a systematic review noted that lower-extremity injuries generally constitute more than 50% of all injuries in rugby sevens players, with anterior cruciate ligament sprains of the knee and lateral ligament sprains of the ankle being the most common injury types [13]. This pattern may be related to the specific biomechanical demands of rugby sevens. Owing to the rapid pace of play and large playing area, athletes are required to perform frequent sudden stops, changes in direction, and high-speed collisions [2]. As the body’s primary weight-bearing and force-generating joints, the knees and ankles are thought to be subjected to continuous repetitive impact and torsional loads. Thus, even a slight delay in neuromuscular control can cause passive stabilizers (e.g., the ACL) to exceed their load tolerance, leading to injury [2]. To date, no published reports have described site-specific injury incidence rates per 1000 h of exposure among Chinese rugby sevens players. Moreover, this study helps address this gap by reporting rates of 0.15 per 1000 training hours for the knee and 0.16 for the ankle.

The higher prevalence of knee and ankle injuries in female athletes may be due to both intrinsic biomechanical deficits and extrinsic training-related factors. Several mechanisms could underlie this disparity. Biomechanically, female athletes have a higher prevalence of increased Q-angle and bilateral Q-angle asymmetry (13.2% vs. 4.2%), according to a 20-year cross-sectional study [22]. Such abnormal alignment may disrupt force transmission and increase patellofemoral and medial knee loading. This could be a key biomechanical risk factor for lower-extremity injury. Females are also reported to have greater knee ligamentous laxity and inferior dynamic joint stability. These characteristics may predispose them to excessive anterior tibial translation and rotational displacement during high-demand movements including running, jumping, and abrupt deceleration, which could further increase the risk of anterior cruciate ligament injury [23]. Notably, female athletes aged >18 years accumulated more annual training volume than their male counterparts. Under equivalent mechanical loading, they typically experienced faster neuromuscular fatigue and greater loss of muscle strength. This may further impair dynamic joint control and amplify the effects of inherent anatomical vulnerabilities.

Notably, our findings demonstrate that female athletes aged >18 years accumulated a greater annual training volume than their male counterparts; under equivalent mechanical loading, female athletes typically experienced faster neuromuscular fatigue and greater attenuation of muscle strength, which further impairs dynamic joint control and amplifies the adverse biomechanical consequences of inherent anatomical and ligamentous vulnerabilities. Collectively, the synergistic interaction between heightened training exposure, reduced physiological tolerance to fatigue, and inherent biomechanical deficits in lower-extremity alignment and dynamic stability may contribute to the disproportionately high risk of knee and ankle injuries in female rugby sevens athletes [23]. We also detected a relatively high incidence of shoulder injuries in this study, accounting for 12.7% of all injury cases and ranking as the third most prevalent injury site. These findings are consistent with previous reports indicating that the shoulder is highly susceptible to contact-related injuries in rugby, especially during competitive matches [24,25]. Biomechanically, the shoulder joint is considered inherently unstable due to its unique anatomical structure, which features a shallow glenoid fossa and relies heavily on dynamic soft-tissue stabilization.

As a central hub for force generation and transmission, the shoulder may be subjected to excessive axial, shear, and torsional forces during high-speed collisions [9]. This could explain its high vulnerability. Biomechanically, the shoulder joint is inherently unstable because of its anatomy. It has a shallow glenoid fossa and relies heavily on dynamic soft-tissue stabilization. The elevated shoulder injury risk may be driven by frequent upper-body contact movements, especially tackling and scrummaging. The shoulder is the primary impact point for tacklers. Ball carriers also face substantial injury risk upon landing, particularly when falling on an outstretched arm or the lateral shoulder region [26].

Age also emerged as a critical modifier of injury patterns, with all age-related analyses statistically adjusted for other variables (sex, height, BMI, and training exposures) to isolate the independent effect of age on injury risk. In this study, athletes aged >18 years exhibited significantly higher injury incidence across key performance-related anatomical regions (knee, ankle, and shoulder) compared with those aged ≤18 years. Although the anatomical distribution of injuries was comparable between age groups (with lower-extremity injuries remaining predominant), the overall injury burden was markedly lower in athletes aged ≤18 years. Biomechanically, athletes aged ≤18 years have incompletely mature musculoskeletal structures, making growth plates vulnerable to overloading. However, their lower injury rate may be largely explained by reduced contact intensity, slower play tempo, and lower annual training exposure compared with older athletes. For upper-extremity injuries, shoulder injuries were the most common across both age groups, yet adolescent athletes demonstrated a markedly lower overall upper-extremity injury rate, attributable to diminished physical contact exposure in junior competitions [27,28]. The elevated injury incidence across both upper and lower extremities in adult athletes may reflect greater physical demands, accelerated movement pace, intensified collision exposure, and higher annual training volume in adult-level training and competition [8,25]. Excessive training loads could further precipitate muscle fatigue and degraded technical execution, synergistically increasing injury risk [29]. After adjustment, age remained independently associated with injury incidence, suggesting that annual training volume acts as a partial mediator rather than a full confounder in the age–injury relationship. These findings indicate that age modulates injury risk not only through physiological maturation but also by altering training exposure intensity and mechanical loading patterns.

## 7. Limitations

This study has several limitations. First, the retrospective cross-sectional design based on self-reported injury data is inherently prone to recall bias, as participants may underreport minor injuries or inaccurately recall injury timing, severity, and context over the 12-month period. Although standardized written instructions for the anatomical reference images were provided, no formal standardized training was administered. Moreover, the investigated data with the lack of formal medical assessment did not include injury type (gradual-onset or acute-onset) and nature (e.g., sprain or fracture). This study was restricted to training-related injuries only, with no investigation of match injuries. Playing positions of the participants were also not investigated. Second, the exclusion of current injuries may introduce selection bias, as athletes with acute ongoing injuries were not enrolled, which may underestimate the overall injury burden in the general rugby population. Third, the 18-year age cutoff was selected based on the standard youth/adult competitive classification in Chinese rugby; however, sensitivity analyses using alternative cutoffs (16 or 19 years) were not performed, which may reduce the robustness of age-related findings. Fourth, no objective training load data (GPS, heart rate, contact intensity) or biomechanical metrics were collected, which precluded quantification of the dose–response relationship between training load and injury. Additionally, the small sample of injured adult female athletes (n = 19) reduced statistical power and generalizability for this subgroup.

Future prospective cohort studies should use wearable sensors (GPS, accelerometers) to quantify real-time training load [10,23], improve sample representativeness via stratified sampling, conduct age-cutoff sensitivity analyses, and integrate biomechanical testing to clarify injury mechanisms [30,31].

## 8. Conclusions

The findings of this study indicate that, among Chinese rugby sevens players aged 14 to 24 years, lower-extremity injuries (ankle and knee) are most common in training. Training injury incidence is higher in players aged over 18 years than in those aged 18 years or younger, and female players, especially >18 years, bear the highest burden. This study investigates the high-risk anatomical regions in rugby and identifies rugby sevens players aged >18 years as priority areas requiring attention, providing scientific data for developing targeted injury prevention strategies.

## Figures and Tables

**Table 1:** Basic characteristics of all participants by sex.

Variable	Male (n = 274)	Female (n = 140)	*p* Value
	Mean (SD)	Mean (SD)	
Age (years)	18.5 (2.6)	19.3 (2.0)	**<0.001**
Height (m)	1.8 (0.1)	1.7 (0.1)	**<0.001**
Weight (kg)	79.9 (11.5)	62.4 (8.4)	**<0.001**
BMI (kg/m^2^)	24.3 (2.9)	22.2 (2.4)	**<0.001**
Years of experience	4.5 (3.6)	4.9 (2.7)	0.166
Training times per week	7.2 (2.6)	7.4 (3.0)	0.393
Training hours per session	2.5 (0.9)	2.3 (0.7)	0.050
Training hours per week	17.8 (8.8)	17.5 (9.6)	0.754
Training hours a year	854.2 (424.1)	839.6 (462.8)	0.754

Values represent mean (standard deviation); bold *p*-value indicates a significant difference between groups.

**Table 2:** Basic characteristics of male and female participants stratified by age.

Variable	Male			Female		
	≤18 (n = 146)	>18 (n = 128)	*p* Value	≤18 (n = 55)	>18 (n = 85)	*p* Value
Age (years)	16.5 (1.3)	20.8 (1.5)	**<0.001**	17.3 (0.9)	20.5 (1.5)	**<0.001**
Height (m)	1.8 (0.1)	1.8 (0.1)	0.267	1.7 (0.0)	1.7 (0.1)	0.480
Weight (kg)	77.1 (11.0)	83.0 (11.6)	**<0.001**	60.8 (7.2)	63.4 (8.9)	0.053
BMI (kg/m^2^)	23.5 (3.0)	25.1 (2.6)	**<0.001**	21.8 (2.3)	22.5 (2.4)	0.073
Years of experience	3.3 (3.4)	5.7 (3.3)	**<0.001**	3.9 (3.01)	5.6 (2.3)	**<0.001**
Training times per week	7.8 (2.6)	6.5 (2.5)	**<0.001**	8.4 (3.1)	6.8 (2.8)	**0.003**
Training hours per day	2.8 (0.9)	2.1 (0.7)	**<0.001**	2.5 (0.6)	2.2 (0.7)	**0.014**
Training hours per week	21.4 (8.3)	13.6 (7.5)	**<0.001**	20.7 (9.1)	15.4 (9.4)	**<0.001**
Training hours a year	1028.9 (397.4)	655.0 (362.3)	**<0.001**	993.0 (438.3)	740.3 (453.4)	**<0.001**

Values represent mean (standard deviation); bold *p*-value indicates a significant difference between groups.

**Table 3:** Distribution of rugby-related injuries among all participants.

Site	Total (n = 414)	Male			Female		
		≤18 (n = 146)	>18 (n = 128)	Total (n = 274)	≤18 (n = 55)	>18 (n = 85)	Total (n = 140)
Knee	53 (18.7)	13 (16.9)	17 (19.3)	30 (18.2)	5 (17.2)	18 (20.2)	23 (19.5)
Wrist	20 (7.1)	7 (9.1)	7 (8.0)	14 (8.5)	2 (6.9)	4 (4.5)	6 (5.1)
Ankle	57 (20.1)	12 (15.6)	18 (20.5)	30 (18.2)	7 (24.1)	20 (22.5)	27 (22.9)
Shoulder	36 (12.7)	10 (13.0)	11 (12.5)	21 (12.7)	1 (3.5)	14 (15.7)	15 (12.7)
Head	6 (2.1)	0 (0.0)	4 (4.6)	4 (2.4)	1 (3.5)	1 (1.1)	2 (1.7)
Hip	3 (1.1)	2 (2.6)	0 (0.0)	2 (1.2)	0 (0.0)	1 (1.1)	1 (0.9)
Lower back	32 (11.3)	9 (11.7)	6 (6.8)	15 (9.1)	4 (13.8)	13 (14.6)	17 (14.4)
Chest	7 (2.5)	2 (2.6)	0 (0.0)	2 (1.2)	2 (6.9)	3 (3.4)	5 (4.2)
Face	10 (3.5)	4 (5.2)	3 (3.4)	7 (4.2)	1 (3.5)	2 (2.3)	3 (2.5)
Elbow	3 (1.1)	0 (0.0)	1 (1.1)	1 (0.6)	1 (3.5)	1 (1.1)	2 (1.7)
Palm/finger	24 (8.5)	11 (14.3)	8 (9.1)	19 (11.5)	2 (6.9)	3 (3.4)	5 (4.2)
Other lower extremity	32 (11.3)	7 (9.09)	13 (14.8)	20 (12.12)	3 (10.34)	9 (10.11)	12 (10.17)
Total	283 (100.0)	77 (100.0)	88 (100.0)	165 (100.0)	29 (100.0)	89 (100.0)	118 (100.0)

Values represent cases (%).

**Table 4:** Injury rates per 1000 training hours by anatomical site among all participants.

Site	Total	95% CI	Male	95% CI	Female	95% CI	≤18	95% CI	>18	95% CI
Knee	0.15	[0.11, 0.19]	0.13	[0.08, 0.17]	0.20	[0.12, 0.28]	0.09	[0.05, 0.13]	0.24	[0.16, 0.32]
Wrist	0.06	[0.03, 0.08]	0.06	[0.03, 0.09]	0.05	[0.01, 0.09]	0.04	[0.02, 0.07]	0.07	[0.03, 0.12]
Ankle	0.16	[0.12, 0.20]	0.13	[0.08, 0.17]	0.23	[0.14, 0.32]	0.09	[0.05, 0.13]	0.26	[0.18, 0.34]
Shoulder	0.10	[0.07, 0.14]	0.09	[0.05, 0.13]	0.13	[0.06, 0.19]	0.05	[0.02, 0.09]	0.17	[0.10, 0.24]
Head	0.02	[0.00, 0.03]	0.02	[0.00, 0.03]	0.02	[0.00, 0.04]	0.00	[0.00, 0.01]	0.03	[0.00, 0.06]
Hip	0.01	[0.00, 0.02]	0.01	[0.00, 0.02]	0.01	[0.00, 0.03]	0.01	[0.00, 0.02]	0.01	[0.00, 0.02]
Lower back	0.09	[0.06, 0.12]	0.06	[0.03, 0.10]	0.14	[0.08, 0.21]	0.06	[0.03, 0.10]	0.13	[0.07, 0.19]
Chest	0.02	[0.01, 0.03]	0.01	[0.00, 0.02]	0.04	[0.01, 0.08]	0.02	[0.00, 0.04]	0.02	[0.00, 0.04]
Face	0.03	[0.01, 0.05]	0.03	[0.01, 0.05]	0.03	[0.00, 0.05]	0.02	[0.00, 0.05]	0.03	[0.00, 0.06]
Elbow	0.01	[0.00, 0.02]	0.00	[0.00, 0.01]	0.02	[0.00, 0.04]	0.00	[0.00, 0.01]	0.01	[0.00, 0.03]
Palm/finger	0.07	[0.04, 0.10]	0.08	[0.04, 0.12]	0.04	[0.01, 0.08]	0.06	[0.03, 0.10]	0.07	[0.03, 0.12]
Other lower extremity	0.09	[0.06, 0.12]	0.09	[0.05, 0.12]	0.10	[0.04, 0.16]	0.05	[0.02, 0.08]	0.15	[0.09, 0.21]
Total	0.80	[0.71, 0.90]	0.70	[0.60, 0.81]	1.00	[0.82, 1.19]	0.52	[0.42, 0.62]	1.21	[1.03, 1.38]

95% CI: 95% confidence interval.

**Table 5:** Injury rates per 1000 training hours by anatomical site in male and female participants by age.

Site	Male				Female			
	≤18 (n = 146)	95% CI	>18 (n = 128)	95% CI	≤18 (n = 55)	95% CI	>18 (n = 85)	95% CI
Knee	0.09	[0.04, 0.13]	0.20	[0.11, 0.30]	0.09	[0.01, 0.17]	0.29	[0.15, 0.42]
Wrist	0.05	[0.01, 0.08]	0.08	[0.02, 0.15]	0.04	[0.01, 0.09]	0.06	[0.00, 0.13]
Ankle	0.08	[0.03, 0.13]	0.21	[0.12, 0.31]	0.13	[0.03, 0.22]	0.32	[0.18, 0.46]
Shoulder	0.07	[0.03, 0.11]	0.13	[0.05, 0.21]	0.02	[0.00, 0.05]	0.22	[0.11, 0.34]
Head	0.00	[0.00, 0.00]	0.05	[0.00, 0.09]	0.02	[0.02, 0.05]	0.02	[0.00, 0.05]
Hip	0.01	[0.00, 0.03]	0.00	[0.00, 0.00]	0.00	[0.00, 0.00]	0.02	[0.00, 0.05]
Lower back	0.06	[0.02, 0.10]	0.07	[0.01, 0.13]	0.07	[0.00, 0.15]	0.21	[0.09, 0.32]
Chest	0.01	[0.00, 0.03]	0.00	[0.00, 0.00]	0.04	[0.00, 0.09]	0.05	[0.00, 0.10]
Face	0.03	[0.00, 0.05]	0.04	[0.00, 0.08]	0.02	[0.00, 0.05]	0.03	[0.00, 0.08]
Elbow	0.00	[0.00, 0.00]	0.01	[0.00, 0.04]	0.02	[0.00, 0.05]	0.02	[0.00, 0.05]
Palm/finger	0.07	[0.03, 0.12]	0.10	[0.03, 0.16]	0.04	[0.00, 0.09]	0.05	[0.00, 0.10]
Other lower extremity	0.05	[0.01, 0.08]	0.16	[0.07, 0.24]	0.05	[0.00, 0.12]	0.14	[0.05, 0.24]
Total	0.51	[0.40, 0.63]	1.05	[0.83, 1.27]	0.53	[0.34, 0.72]	1.41	[1.12, 1.71]

95% CI: 95% confidence interval.

**Table 6:** Logistic regression analyses of the association of the incidence of rugby-related injuries and variables.

Variable	Model 1 OR (95% CI)	*p*	Model 2 OR (95% CI)	*p*	Model 3 OR (95% CI)	*p*
Gender						
Female	1.00		1.00		1.00	
Male	1.28 (0.83–1.97)	0.263	1.94 (0.96–3.90)	0.064	2.03 (1.00–4.14)	0.050
Age (yrs)	1.15 (1.05–1.26)	<0.002	1.12 (1.02–1.23)	0.017	1.14 (1.03–1.27)	0.013
Height (cm)	-		5.61 (0.13–244.72)	0.371	6.16 (0.14–278.51)	0.350
BMI (kg/m^2^)	-		1.07 (0.99–1.16)	0.078	1.07 (0.99–1.16)	0.080
Training years of experience	-		-		0.99 (0.93–1.06)	0.826
Training hours daily	-		-		1.37 (0.98–1.91)	0.062
Training hours weekly	-		-		0.98 (0.95–1.01)	0.266

BMI: body mass index, calculated by weight and height; OR: odds ratio; and 95% CI: 95% confidence interval.

## Data Availability

The datasets used and/or analyzed during the current study are available from the corresponding author upon reasonable request.
